# From Ocean to Medicine: Harnessing Seaweed’s Potential for Drug Development

**DOI:** 10.3390/ijms25020797

**Published:** 2024-01-08

**Authors:** João Cotas, Silvia Lomartire, Ana M. M. Gonçalves, Leonel Pereira

**Affiliations:** 1 Marine Resources, Conservation and Technology, Marine Algae Lab, CFE—Centre for Functional Ecology: Science for People & Planet, Department of Life Sciences, University of Coimbra, 3000-456 Coimbra, Portugal; jcotas@uc.pt (J.C.); silvia.lomartire@uc.pt (S.L.); amgoncalves@uc.pt (A.M.M.G.); 2 Department of Biology and CESAM, University of Aveiro, 3810-193 Aveiro, Portugal

**Keywords:** seaweed-derived compounds, drug discovery, bioactive compounds, formulation strategies, pharmacological activities, drug development

## Abstract

Seaweed, a miscellaneous group of marine algae, has long been recognized for its rich nutritional composition and bioactive compounds, being considered nutraceutical ingredient. This revision delves into the promising role of seaweed-derived nutrients as a beneficial resource for drug discovery and innovative product development. Seaweeds are abundant sources of essential vitamins, minerals, polysaccharides, polyphenols, and unique secondary metabolites, which reveal a wide range of biological activities. These bioactive compounds possess potential therapeutic properties, making them intriguing candidates for drug leads in various medical applications and pharmaceutical drug development. It explores their pharmacological properties, including antioxidant, anti-inflammatory, antimicrobial, and anticancer activities, shedding light on their potential as therapeutic agents. Moreover, the manuscript provides insights into the development of formulation strategies and delivery systems to enhance the bioavailability and stability of seaweed-derived compounds. The manuscript also discusses the challenges and opportunities associated with the integration of seaweed-based nutrients into the pharmaceutical and nutraceutical industries. Regulatory considerations, sustainability, and scalability of sustainable seaweed sourcing and cultivation methods are addressed, emphasizing the need for a holistic approach in harnessing seaweed’s potential. This revision underscores the immense potential of seaweed-derived compounds as a valuable reservoir for drug leads and product development. By bridging the gap between marine biology, pharmacology, and product formulation, this research contributes to the critical advancement of sustainable and innovative solutions in the pharmaceutical and nutraceutical sectors.

## 1. Introduction

Natural compounds have traditionally provided significant contributions to pharmacology. Natural compounds, however, pose hurdles for drug development, such as technical impediments to screening, isolation, characterization, and optimization, which has contributed to a downturn in their pursuit. However, with the emergence of new diseases, ineffectiveness of conventional therapies, and the development of multidrug resistance infections are damaging the human health and the normal therapies efficacy and efficiency. Furthermore, natural compounds are being targeted to develop new drugs that can be key to enhance the human health [1,2,3,4]. However, from the discovery until the real usage there is a long road to do [4,5].

Oceans, which represents 75% of the planet Earth, can be key factor to find new potential drugs to be applied to treat human diseases and illness. Although, the marine organism’s potentiality into pharmacological area is scarce, being developed in the recent years [5]. The marine habitat provides a vast and diversified source of novel drugs to tackle major ailments such as cancer, infections, cardiovascular diseases, and malaria. Antibacterial, immunomodulator, antifungal, anti-inflammatory, anticancer, antimicrobial, neuroprotective, analgesic, and antimalarial effects are being investigated in these aquatic creatures. They are widely employed in the research of novel drugs all around the world [1,2,3,4,5], where seaweeds are among the most important organisms being studied.

Seaweed is generally used in a variety of industries (food, nutraceuticals, cosmetics, pharmaceuticals), but its increased use, as well as regulation and approvals to allow its consumption, will be dependent on future generations and will evolve over the coming years, conditional on how research and market request change. On the other hand, there must be a development in seaweed cultivation, processing, and procedures to boost their efficiency and efficacy. Seaweed may be used into an ample range of goods; it is a creative approach for research and manufacturing to capitalize on the potential of these organisms in many sectors [6].

There is currently an emphasis on sustainability to aid the environment, and seaweed is an “agricultural” system with low related costs since fertilizers, feed, and pesticides are not required as much as in other systems. Aquaculture and macroalgae systems are already displacing other businesses in several countries, and people’s economic situations have improved. Despite the benefits listed above, there are some hazards linked with seaweed. Due to environmental changes that directly impact the chemical composition of the product, it is challenging to manage the seaweed bioactive compounds, as well as the quality of the final product. On the other hand, because seaweed differ so much between species, it is impossible to evaluate them in general terms [7,8].

For instance, brown seaweed typically contains phlorotannins and fucoxanthins in concentrations ranging from 12 to 250 mg/g d.w., influenced by factors like location, time of year, and weather conditions [9]. In a Japanese study, *Saccharina japonica* (formerly *Laminaria japonica*) exhibited concentrations of 14.9 mg/g d.w., while *Undaria pinnatifida* showed 5.9 mg/g d.w. [10]. Montero et al. [11] examined *Sargassum muticum* (Phaeophyceae) samples from different spots along the European Atlantic coast, revealing phlorotannin concentrations ranging from 1.4 mg/g d.w. to 94.0 mg/g d.w. The analysis also considered various brown seaweed species from different geographic origins, underscoring the challenges in result comparison due to variations in experimental conditions like solvent, solid-solvent ratio, temperature, and extraction time [12]. This emphasizes the crucial need for standardized protocols.

Another question is whether the seaweed compounds and organoleptic properties can be altered during compound extraction and purification. As a result, there must be a strong interest in promoting potential pharmaceutical safety and security. The overall effects on the chemical structure of the seaweed components (principally, molecular weight, bioactivity, and toxicity) are a key factor to develop new seaweed-based drugs. Thus, it is critical to endorse rigorous analysis of the final product as well as the development of a certifying system to promote seaweed compounds as drugs [1,6,13]. However, seaweeds demonstrate high biotechnological potential for drug development, due to their natural plasticity, compound richness and the possibility to work their composition to obtain specific compounds [8,14].

Seaweeds are potential sources of high biotechnological interest due to the synthesis of a wide range of chemicals with diverse biological functions. However, there is a critical requirement for management approaches for a long-term strategy to use marine organisms as a source of bioactive compounds. Various seaweed species can produce a wide range of secondary compounds, which play significant environmental roles as protection and/or signal molecules and are also of biotechnological interest. Furthermore, intriguing insights into the bioactivities of seaweed extracts, fractions, and isolated molecules have fueled the research and development of seaweed-based pharmaceuticals with commercial potential in recent years. Some of the molecules identified from seaweeds that have garnered the most interest from the pharmaceutical industry for the research and development of novel pharmaceuticals are sulfated polysaccharides (anticoagulants, anticancer, and antivirals), dieckol (cardiovascular diseases) and kahalalide F (anticancer and anti-aids compounds) [1,2,3,15,16]. Nevertheless, the road to seaweeds achieve their full potential is still very far due to the long road and complexity between the identification of the biotechnological potential up to the drug development and approval.

Thus, this review will focus on the critical point of understanding the seaweed compound potential into pharmaceutical drugs. Focusing, in the next steps after compound screening hit, due to be high number of reviews about potential seaweed compounds. But little is known after that and the long road into the clinical assays. There is a need to critical think after the positive, mostly the compound stability, reproducibility, seaweed variability, certification assays, and the pharmaceutical approval.

## 2. Methodology

For this critical literature review, we carried an exploratory search using the Pubmed database from the National Center for Biotechnology Information (NCBI) of the United States of America (available at https://pubmed.ncbi.nlm.nih.gov/) (accessed on 15 December 2023) to disclose the protocol for drug approval and their information on the seaweed drug approval.

Data were collected from online databases, mainly Web of Science, Google Scholar, Science Direct, and Scopus, considering research articles, books, chapters, news, websites, and reviews. The selected topics included the following combinations: seaweed, macroalgae, seaweed compound and drug potential, cultivation, security, safety, extraction, isolation, chemical safety, pharmacological, drug potential, cultivation, security, safety, extraction, isolation, chemical safety, pharmacological, medicinal, bioactivity, organic synthesis, regulation, formulation, pharmacodynamics, pharmacokinetics, approved drugs, delivery system, and there more keywords used (we tried to obtain the maximum data available). Also, during this work, we target one of the considered critical lacks the literature that was observed when gathering information which consists of the potential application of seaweed-based compound until an approved drug, and their restrictions and potential solutions. Moreover, during the writing of the manuscript, other keywords more specific was searched, such as seaweed preclinical compound, drug approved drugs in google and news sites, chemical systems/methods to alter natural structures into drugs and seaweed mechanism of action. We attempted to obtain the maximum data with scientific support to be analyzed and based in the literature reviews. The study focused linking the two major key points that are academic research and discovery studies to the potential exploitation of the specialized industry.

## 3. Seaweed Diversity and Bioactive Compounds

Seaweeds, also recognized as macroalgae, are multicellular photosynthetic plant-like organisms found mostly in seas and oceans. They are a commercially significant natural resource that is abundantly available, and their potential as food-grade feedstock should not be overlooked, particularly in the context of bioactive compound forecasts [17]. Furthermore, because seawater covers around 70% of the Earth’s surface, sustainable cultivation and harvesting of seaweeds is feasible, as they typically have strong growth rates, do not compete with agriculture for land and drinking water, and emit little or no greenhouse gases [18].

Seaweeds have evolved mechanisms to withstand biotic threats such as bacteria, viruses, and fungal infections over millions of years. Seaweeds have evolved to exist under abiotic environmental and stress circumstances that are varied, harsh, and unfriendly, such as temperature and salinity changes, environmental pollutants, or UV light exposure from sessile plants. As a result, these organisms can produce a wide range of secondary bioactive chemicals, including pigments, vitamins, phenolic compounds, sterols, and other bioactive molecules. In addition, they create amino acids and proteins, saturated and unsaturated fatty acids, and polysaccharides, all of which are directly engaged in the development, growth, and reproduction of organisms, allowing them to execute physiological functions [19]. Furthermore, there are various environmental intrinsic and extrinsic variables that have a substantial influence on seaweed compounds yield and quality, such as season, chemical pollution, maturity, microbiome, sunlight, pH. Because of these ecological effects, there is uncertainty in the yield and bioactive potential and production of seaweed bioactive compounds from wild and cultivated seaweed, making them a problematic raw element for industrial utilization even using established and secure extraction methods [8,16].

Despite this, isolated seaweed chemicals have been examined and found to have anti-bacterial, anti-viral, anti-allergic, anti-diabetic, antioxidant, anti-photoaging, anti-pruritic, hepato-protective, hypotension, neuroprotective, and anticancer activities [1,20].

Furthermore, seaweed compounds, such as the brown seaweed polymer alginate, are used as active agents or as secondary compounds for encapsulating or stabilizing the active agent in a range of medicines and novel therapies [21]. However, phenolic compounds, pigments, and polysaccharides are the most studied seaweed compounds in the biomedical field [2]. Although, there is three major compounds from seaweeds that are being key elements into the RD and pharmaceutical units: polysaccharides, phenolic compounds, and pigments. These three types of compounds are the major compound on seaweeds, thus, high quantity and quality. Moreover, these compounds are already used and applied in various industry, being the extraction and isolation very well known. Although, for pharmaceutical area every method and safety need to be carefully analyzed and used, since impurities or a molecular chemical shifting (in the target compound) can damage the human body.

### 3.1. Phenolic Substances

**Figure 1:**
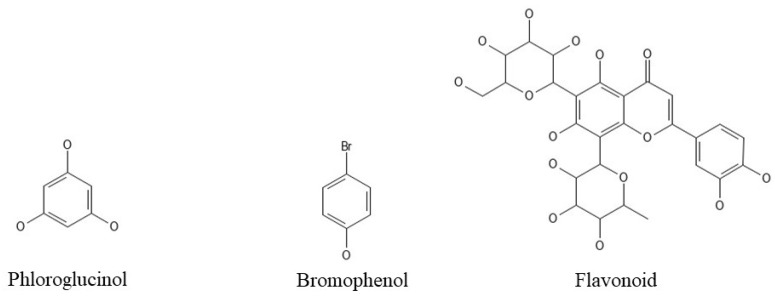
Phenolic compounds structures.

Phenolic compounds (Figure 1) are byproducts of seaweed metabolism. They are a complicated class of water-soluble chemical compounds with a hydroxyl group linked to an aromatic hydrocarbon group. Phenols are classed as basic phenolic compounds based on the number of substituents, which include terpenoids, flavonoids, phlorotannins, bromophenols, and many mycosporine-like amino acids [16,21].

These compounds have a wide range of bioactivities, including anti-tumor, anti-diabetic (commercially available drugs), antiviral, antioxidant, neuroprotective, anti-inflammatory, and sleep-promoting characteristics (for addressing insomnia and other sleep-related disorders) [16].

### 3.2. Pigments

**Figure 2:**
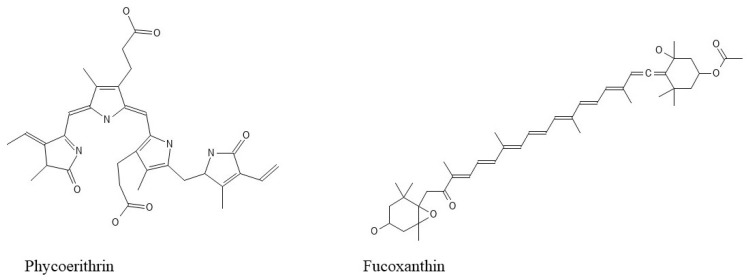
Pigments structures.

As photosynthetic organisms, seaweeds can produce three types of pigments: chlorophylls, carotenoids, and phycobiliproteins. The pigments in seaweed are determined by their color. The green color is caused by the presence of chlorophylls *a* and *b*. The red color is caused by phycobilins such as phycoerythrin and phycocyanin. Brown seaweeds are typically pigmented with chlorophylls a, c1, and c2, b-carotene, and fucoxanthin (Figure 2) [22]. These isolated compounds have antibacterial, anti-inflammatory, neuroprotective, antioxidant, and anti-tumor properties [23]. Furthermore, these compounds are being researched for use as fluorescent indicators in the biomedical field [24].

### 3.3. Polysaccharides

**Figure 3:**
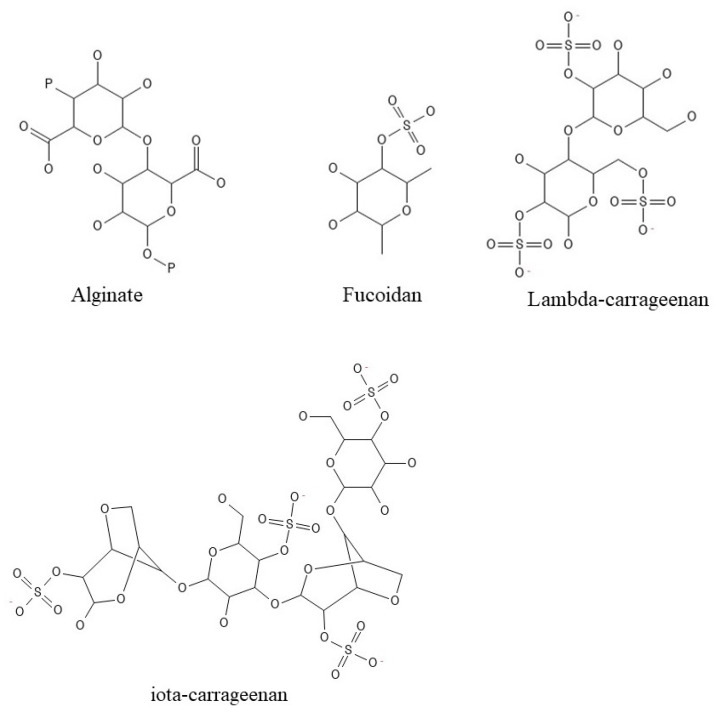
Polysaccharides structures.

Seaweed-derived polysaccharides (Figure 3) with specific structural and functional properties have gotten exceptional investigation interest in the current biomedical field [25].

Scientists and the industry have been developing novel biomaterials for drug delivery, tissue engineering, and wound dressings by leveraging the advantageous inherent qualities of seaweed polysaccharides. These include characteristics that are biologically adjustable, biocompatible, biodegradable, recyclable, and non-toxic [21,25]. Seaweed polysaccharides have a controlled distribution and are therapeutically effective [25].

Especially with alginate, an anionic polymer with low bioactivity compared to fucoidan, an anionic sulfated polysaccharide extracted from brown seaweed with a wide range of bioactivities including anti-inflammatory, anti-oxidative, anticoagulant, and antithrombotic effects [26]. In the past decade, there has been thorough examination of fucoidan for its potential applications in drug and gene delivery systems, along with diagnostic microparticles [26].

Derived from red seaweed, carrageenan has been a widely employed remedy since ancient times for treating coughs and common colds, a usage supported by both in vitro and in vivo tests. The primary basis for this efficacy lies in carrageenan’s capacity to hinder blood platelet aggregation, demonstrating its anticoagulant activity [27,28]. Different carrageenans excel in other demonstrable bioactivities such as anti-tumor, anti-viral, and immunomodulation activities, and their anti-viral properties are commercially exploited [29].

Agar finds application in the biomedical field as a bulking and suspension agent in medicinal solutions and prescription products. Additionally, it is used in capsules and tablets for its anticoagulant and laxative properties. They are also employed to create novel biomedical tools for analysis and characterization [30,31,32].

### 3.4. Other Biomedical Compounds of Interest

There are compounds isolated from seaweed that have received little attention in the literature [33,34,35].

Fatty acids hold promise in contributing substantially to the advancement of novel biomedical solutions for immunomodulation drugs, as well as for the treatment and prevention of various conditions such as neoplastic, ocular, cardiovascular, neurodegenerative, and autoimmune disorders [23,33]. Seaweed sterols are plant and animal hormone precursors with numerous bioactivities, including antioxidants, antivirals, antifungals, and antibacterial properties [36].

Seaweeds may be used to extract a variety of secondary bioactive compounds with significant medicinal and industrial potential. Antifungal, antibiotic, antiviral, contraceptive, anti-inflammatory, anticancer, antioxidant, and anticoagulant effects are among the bioactivities of seaweed metabolites.

Despite the biomedical potential of seaweeds, only a limited number of seaweed compounds are presently utilized in the field of biomedicine [16,37,38,39,40,41]. Because biological interest in seaweed is still relatively young, further research and development are needed to explore additional potential seaweed chemicals for this sector. Extraction efficiency in isolating and enhancing the critical bioactive components, as well as optimal agricultural practices, are necessary to maximize the beneficial utilization of seaweed metabolite activities for human health [39,41].

## 4. Extraction and Characterization of Seaweed-Derivate Compounds

Even with these variations of the seaweed composition, the advancement of modern technology, there it has been feasible to demonstrate the health benefits of seaweeds. In the last several decades, researchers have used in vitro and in vivo tests to demonstrate the antioxidant, antibacterial, antiviral, and antitumor characteristics of seaweed extracts and compounds, with a focus on the isolation and mechanism of action of each molecule (pre-clinical assays). Moreover, the usage of seaweeds is both sustainable and cost-effective, given their ability to thrive in various aquatic habitats and their easy cultivation, mainly relying on sunlight, aeration (whether natural or artificial), and saltwater abundant in nutrients [3].

Although the most important and vital point in seaweed exploitation is the compound extraction and purification, as the compound purity rate it is a key element for a compound when there is the hypothesis to obtain an active ingredient for a pharmaceutical drug.

It is suggested that seaweed be used as a pre-treatment, for instance, employing a washing process is essential to eliminate stones, sand, epiphytes, and other contaminants. Consequently, the algal biomass can be employed in its fresh state, subjected to drying methods such as air drying or aeration at 30–40 °C for 3–5 days, or undergo freeze-drying. The latter is preferred as it safeguards the integrity of biomolecules and enables higher extraction yields [13,16,42,43].

Milling or grinding the algal biomass before the pre-treatment is also recommended to reduce particle size, which increases the area of exposure between the seaweed biomass and the solvent used for extraction. As a result, extraction yield will increase [44,45].

The final stage involves selecting an extraction technique, and there can be considerable variation in methods. Traditional extraction procedures include Soxhlet extraction, solid-liquid extraction, and liquid-liquid extraction. Commonly used organic solvents, such as hexane, petroleum ether, cyclohexane, ethanol, methanol, acetone, benzene, dichloromethane, ethyl acetate, and chloroform, are applied in these processes. However, it is essential that the solvent is non-toxic, safe, and cost-effective. [44,45]. After employing the extraction method, it is crucial to isolate the separated and quantified target component. Various approaches may be employed depending on the nature of the material to be separated. Factors such as the seaweed constituents’ raw source, the extraction and purification techniques applied, the particle size of the sample, storage conditions, and the presence of interfering components in extracts can all influence the results and bioactivities [16,46,47]. According to a recent study, the same seaweeds landed in different intertidal zones, and most seaweeds could not withstand the extremely bad conditions of low DO, low salinity, or abundant feeding. When seaweeds survived in such harsh environments, they often showed enhanced antioxidant activity. Seaweeds that survived in poor conditions appeared to have a higher total phenol content than other similar varieties settled in mild conditions; additionally, antibacterial activity in seaweeds that survived in very poor conditions of low DO, low salinity, or rich nutrition was not significantly different from those settled in moderate conditions. Therefore, the final bioactivity is influenced by biotic and abiotic factors, and before promoting natural compounds as valid elements for pharmacological formulations, a deep investigation needs to be performed, for each compound [48].

Preparative chromatography methods, including column chromatography, high-pressure liquid chromatography (HPLC), and thin-layer chromatography (TLC), are currently employed for extracting components from seaweed (Table 1). These chromatographic techniques have been well-established for separating, isolating, purifying, identifying, and quantifying various seaweed constituents, including vitamins, proteins, phenolic compounds, and pigments [49,50,51].

A Quadrupole Time of Flight High-Resolution Mass Spectrometer (Q-TOF-HRMS) was employed to conduct metabolomics profiling through mass spectrometry, aiming to identify compounds based on precise mass measurements through database searches.

In recent times, the Quadrupole Time of Flight High-Resolution Mass Spectrometer (QTOF-HRMS) has become a preferred tool for accurately measuring mass in small molecules, handling multiple charge ions, distinguishing isobaric species, annotating non-target compounds, and determining elemental composition without compromising sensitivity. Various tools, such as the Molecular Formula Generation (MFG) software from Agilent Technologies, leverage mass accuracy and spectra to minimize ambiguity, providing a list of potential molecular formulas. The availability of a database library further streamlines the annotation of compounds using accurate mass data. Mass fragmentation aids in enhancing the characterization of the identified compounds [52].

Until recently, Infrared (IR) spectroscopy stood out as the predominant vibrational technique employed to investigate the chemical composition of polyphenols (phycocolloids). This method boasts two primary benefits: it demands only small sample quantities (in milligrams), and it is a noninvasive approach known for its dependable accuracy [53].

Vibrational spectroscopy, specifically Fourier-Transform Infrared (FT-IR) and Raman spectroscopy, holds significant potential for the quantitative analysis of biopolymers. Both Raman and FT-IR spectroscopy offer non-invasive and non-destructive capabilities for real-time routine analysis. Moreover, Raman spectroscopy exhibits notable advantages in analyzing crystalline phases of drugs. In comparison to FT-IR, Raman spectroscopy is more sensitive, enabling the detection of polymorphic forms of drugs with detailed spectral information of trace elements in situ and with high resolution. Raman spectroscopy remains excellent for discriminating between different polymorphs and pseudo-polymorphs of substances. On the other hand, FT-IR spectroscopy appears to be less sensitive in determining particle surface morphology after solid-state transitions induced by compression [54].

Due to the high cost of these procedures, they are still in the early stages of efficiently exploiting seaweed compounds, though they are being studied for future applications in pharmaceutics. Unlike polysaccharides, which are easily to extract, and the advancement of new extraction technologies allows the extraction to be quicker, environmentally sustainable, eco-friendly, reproducible, and more efficient compared to traditional methods which involved many organic solvents and are more time consuming [55,56].

Ultrasound and microwave-assisted extraction are currently low-cost, large-scale methods considered valid to extract seaweed polysaccharides in an eco-friendly way and obtaining active polysaccharides [16,44,45].

## 5. Therapeutic Potential and Safety Concerns

Seaweeds have been employed in food and medicine in the past, especially in traditional Asian medicine, predating a comprehensive understanding of the mechanisms of action of their constituents [3].

Due to their potential health advantages, encompassing antioxidant, anti-inflammatory, antibacterial, antidiabetic, antiviral, anti-inflammatory, anticoagulant and anti-cancer properties, these chemicals have garnered significant attention. However, concerns have been expressed regarding their safety and toxicity, particularly in relation to their extraction and isolation methods, which can influence their relative safety and toxicity. Given the diverse chemical structure and impurities, there is a necessity to standardize the procedure from extraction to safety/toxicity assays [57,58,59,60].

Several studies have been conducted to investigate the safety and potential toxicity of seaweed compounds. In general, the available evidence indicates that these compounds are safe for consumption in moderate amounts. Nevertheless, there are reservations regarding their potential toxicity when taken in high doses [57]. Algae possess the capability to absorb substantial quantities of heavy metals, including mercury, cadmium, lead, copper, and thallium. The organic compound Methylmercury, upon entering the human body, can induce chronic toxic reactions, potentially causing significant pain or organ failure. In the field of algal food safety testing technology, it is vital to create tools capable of measuring mercury levels in algae-based food. For instance, the development of a quick and straightforward detection kit to assess the toxicity of water contaminated with heavy metals and a method for promptly determining mercury levels in spirulina health supplements would be advantageous. As a result, industries engaged in food production should actively monitor variations in mercury content in algal food during various stages of preparation, processing, and preservation. This approach aids in a more effective assessment and control of mercury levels in seaweed products. Additionally, insufficient iodine intake can result in goiter, characterized by neck enlargement, and reduced thyroid hormone levels, potentially causing damage to the central nervous system. In children, this deficiency can lead to intellectual disabilities and slow reaction times, impacting normal growth and development. Simultaneously, inadequate iodine intake can decrease thyroid hormone synthesis and secretion, leading to a reduction in human metabolism, particularly in women [61].

The bioavailability of seaweed has been a relatively understudied area so far, necessitating further research and exploration. Most investigations into the pharmacological and biological bioavailability of seaweed compounds have relied on mouse models. Both animal and in vitro studies have demonstrated the protective effects of seaweed compounds against various illnesses. Consequently, there is a pressing need for new research aimed at comprehensively understanding their bioavailability in humans, specifically focusing on the proportion of the chemical that reaches the human circulatory system and exerts an active effect.

Moreover, a more thorough exploration of pharmacokinetics is essential to fully grasp the potential of seaweed compounds in in vivo models. This is particularly crucial due to a general lack of information, as the therapeutics currently in use lack comprehensive public data on this topic [6,16,62,63,64].

While phenolic compounds exhibit a diverse array of biological actions, the question of a safe dosage requires attention. Phenolic substances demonstrate bimodal pharmacological effects, being beneficial at low doses but potentially toxic at higher concentrations. However, a significant portion of current research on the adverse effects of phenolic chemicals is centered on cell studies and animal models, with limited human trials [62]. The concern over the potential contamination of marine organisms by heavy metals, such as lead, mercury, and cadmium, is a primary consideration. Accumulation of these heavy metals in the tissues of marine organisms can pose health risks if consumed in large quantities. Hence, it is crucial to source marine polyphenol supplements from reputable suppliers to minimize the risk of heavy metal contamination. Another aspect of concern is the potential for allergic reactions to marine polyphenols. Some individuals may have allergies to specific types of marine organisms or their derivatives, leading to adverse reactions. Therefore, before incorporating marine polyphenol supplements into your routine, it is advisable to check for any allergies [65].

Additionally, the effects of marine polyphenols on pregnant and nursing women are not fully understood, and it is recommended to avoid these compounds during these periods [66].

Despite the demonstrated potential health benefits of seaweed compounds, ensuring their safety, and minimizing potential toxicity is paramount. Moderation in consumption and obtaining marine polyphenols from trustworthy sources is advised. Moreover, consulting with a healthcare professional before initiating any new supplement regime is always recommended [67].

### Potential Bioactivities of Seaweeds

Seaweeds compounds possess several interesting biological activities, some of them are listed in Table 2. This thematic has been very well studied and there are various literature reviews based only in the seaweed potential bioactivities [1,2,3,5,15,16,22,28,35,68,69,70,71,72,73,74,75,76,77,78]. Due to its being a developed thematic in various literature reviews, in this study, we will lightly explain the seaweed potential bioactivities.

Antibiotics are among the most significant medicinal used for the treatment of illnesses all over the world. However, the emergence of antimicrobial resistance against a wide range of infections has a detrimental influence on therapeutic success, and the emergence of drug resistance poses a potential threat to patient lives. Therefore, the pursuit of innovative antimicrobials that can withstand resistance becomes crucial. Seaweeds contain secondary biomolecules like the polysaccharide fucoidan, sulfoquinovosyldiacylglycerols, and caulerpin, which exhibit diverse biological activities, including antibacterial effects. The antimicrobial chemicals found in seaweeds primarily serve as components of the seaweed’s natural defense system against invading pathogens [72,79].

Through the stimulation of the host’s immune system or the inhibition of virus multiplication prior to the virus’s entry into host cells, seaweed metabolites can function as antiviral medications. Several viruses, including herpes, lentivirus, influenza, and coronaviruses, can be targeted by seaweeds [80,81]. Polyphenols and sulphated polysaccharides have higher antiviral activity than other seaweed components. Due to their ability to prevent viruses from attaching to the negatively charged surface of host cells, sulphated polysaccharides have virucidal properties. Negatively charged sulphated polysaccharides and positively charged viral glycoproteins can interact to stop the virus from infecting the target cell [80].

For example, sulphated polysaccharides isolated from seaweeds such as galactan, carrageenan, fucoidan, ulvan, alginate, naviculan, and calcium spirulan have been discovered to provide inhibitory effects against cell damage caused by numerous viruses [81]. The researchers discovered that seaweed extract has significant growth potential and has been successfully marketed for antiviral purposes. Consequently, natural polysaccharides derived from seaweed have the potential to be a safer and more effective antiviral medication than manufactured ones [75]. Several vector-borne illnesses might potentially be treated with seaweed-derived substances. Dengue virus illness, which is spread by mosquitos, is one of the most frequent diseases that causes epidemics and kills many people each year, notably in India. The most common dengue virus carriers are *Aedes albopictus* and *Aedes aegypti*. Moreover, the chikungunya virus attaches itself to the surface of the host cell and starts to replicate. Therefore, restricting the attachment and binding of the virus to its host may be a helpful strategy for managing viruses. Using polysaccharides generated from seaweed can effectively prevent some deadly viral infections because they can alter the characteristics of cell surfaces [39].

Seaweed has shown strong antioxidant mechanisms in extremely oxidative conditions. Since seaweed is a photosynthetic organism, it is frequently exposed to light and oxygen, which promotes the production of powerful oxidizing agents like free radicals. The absence of oxidative damage to the thylakoid membranes of the chloroplast, where 301 macroalgal metabolites—including sulphated polysaccharides, polyphenols, unsaturated fats, amino acids, and peptides—is indicative of seaweed’s robust defenses against oxidative agents [82].

The body uses its natural defense mechanisms, the immune system, and the inflammatory response, to deal with wounds, combat infections, and return homeostasis [83].

Prolonged and unnecessary inflammation caused by pathogenic pathogens and necrotic cells, on the other hand, must be regulated to avoid tissue damage [84]. In addition to essential oils, polyunsaturated fatty acids, sulphated polysaccharides, fucoxanthin, alkaloid, and astaxanthin have been linked to anti-inflammatory activities in seaweeds [85,86].

Anyanji et al. [87] found that *Kappaphycus alverazii* (Rhodophyta) has anti-inflammatory action when compared to the commercial antihistaminic drug “Loratadine” in treating asthma symptoms in mice, lowering mucus formation and downregulating proinflammation genes. This might be a potential addition to the helpful effects of seaweed in lowering the symptoms of chronic asthmatic sufferers.

Cancer counts among the leading causes of death worldwide. Efforts to create novel, safe, and effective cancer chemotherapy from natural resources are essential, especially in aquatic environments, since cancer chemotherapy negatively affects neighboring normal cells [88]. Seaweeds may be employed as anticancer drugs because of the existence of particular components, such as carotenoid fucoxanthin and sulphated polysaccharides [2]. Several research have discovered a substantial relationship between seaweeds’ phenolic antioxidant capacity and their anticancer effects [39].

Heparin is the most often used anticoagulant medicine in the biomedical sector for the treatment of thromboembolic illnesses. Thrombocytopenia, a hemorrhagic consequence of heparin, is the most often documented side effect. This needs the development of new antithrombotic drugs [85]. Seaweed polysaccharides have been shown in certain studies to possess anticoagulant properties and to be free of prions or dangerous viruses that are known to contaminate commercial heparins [89]. Seaweed extracts are preferred over commercial heparin because seaweed polysaccharides are likewise safe for cellular digestion. The pharmacological pathway of seaweed polysaccharides is entirely determined by the presence, position, and molecular weight of the sulfate group. Scientific literature has identified fucoidans, phlorotannins, and sulphated polysaccharides produced from brown algae as anticoagulant agents [90].

Despite some encouraging results, most pharmacological studies on seaweeds are still in its early stages, and additional assays and trials are needed. Mostly due to be one hit success and not reproducible assay due to be inherent variability in the seaweed composition.

**Table 2:** Biological activity expressed by seaweed’s compounds.

Seaweeds Compound	Biological Activity	Reference
Fucoidan, sulfoquinovosyldiacylglycerols, caulerpin	Antimicrobial activity	[72,79]
Galactan, carrageenan, fucoidan, ulvan, alginate, naviculan, calcium spirulan	Antiviral activity	[39,75,80,81]
Sulphated polysaccharides, polyphenols, unsaturated fats, amino acids, peptides	Antioxidant activity	[82]
Essential oils, polyunsaturated fatty acids, carrageenan, fucoxanthin, alkaloid, astaxanthin	Anti-inflammatory activity	[85,86,87]
Carotenoid, fucoxanthin, sulphated polysaccharides, phenolic compounds	Anticancer activity	[2,39]
Sulphated polysaccharides, fucoidans, phlorotannins	Anticoagulant activity	[89,90]

## 6. From Seaweed to Pharmaceutical Drug

Metabolites derived from seaweeds are referred to as bioactive chemicals and possible potential drugs. Aside from having pharmacological or toxicological effects on organisms, which has led to their use in the food and pharmaceutical industries, the discovery of novel properties of such compounds has led to a diversification of their applications. However, there is a need to go further into regulations and considerations to observe if a select compound was potential to be exploited in industrial level.

**Figure 4:**
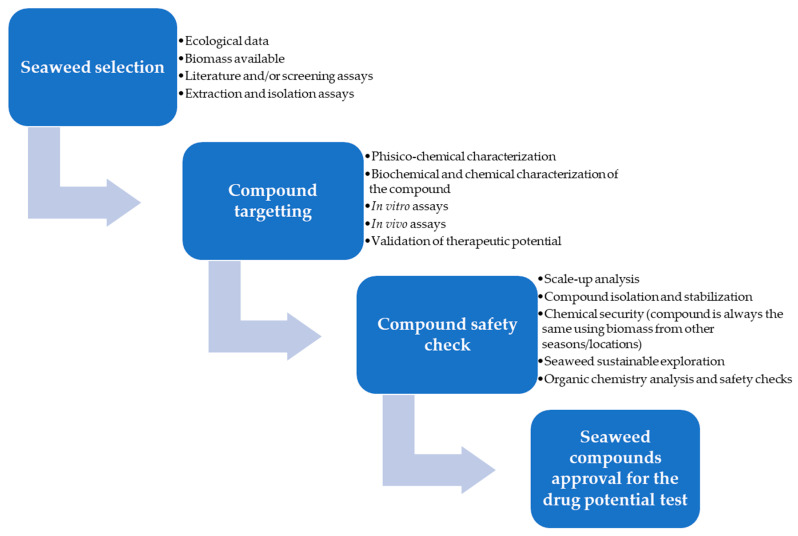
First steps to exploit seaweed compound into the drug clinical phases.

Through the present review, all the steps that lead to certified natural compounds from seaweeds are discussed, starting with the compound sourcing. Figure 4 presents the initial stages in the research of the new seaweed compounds in the discovery and development, this is a keystone to exploit one compound with sustainable and economic point of view.

### 6.1. Seaweed Cultivation

Seaweed farming has given a long-term alternative to depleting natural resources. It has now reached commercial significance in Asia and is gaining interest in Europe. The global need for enormous amounts of seaweed will increase in the next years. Nonetheless, continuous optimization of the culture system is now being carried out to meet this expanding demand, ensuring long-term algal production). Collaboration between academia and the aquaculture industry through research and development (R&D) centers has resulted in the development of research initiatives to find possibilities and technologies to improve the efficiency and productivity of algal aquaculture systems, with the goal of improving their ecological sustainability and suitability for the blue economy [19]. Seaweed farming is typically at the forefront of the sustainable exploitation of seaweed bioactive and targeted chemicals because to its safety and production control since culture would incur additional costs while maintaining safety. However, to fully use this methodology, cultivation methods must be developed from a multidisciplinary standpoint to guarantee the stability and bioactivity of the targeted molecule [8].

To achieve a specific yield and quality of a particular seaweed compound with a high rate of success, preliminary cultivation studies can help control and understand the impact of extrinsic and intrinsic factors, while putting less strain on the wild sea-weed community and lowering production costs. The promise these studies offer, however, suggests that there is still much work to be done before we fully comprehend which factors affect the quantity and quality of phenolic compounds and how to use this information in large-scale production systems. Because phenolic compound bioactivity and production are usually species dependent, the species must be examined beforehand in controlled growth environments. Another method for investigating this topic is to recreate the environmental conditions that resulted in the greatest bioactivity and attempt to collect data for all accessible parameters evaluated and investigated. It may be easier to collect data, but testing the culture process may be more complex. However, if the seaweed is discovered in a variety of geolocations with variable results, this strategy may be an appropriate preliminary inquiry prior to the seaweed cultivation test [8,91,92,93,94]. Thus, this can be an important key for new seaweed-based drugs.

### 6.2. Extraction and Organic Synthesis

However, there are several current trends that use natural compounds with chemical alterations to stabilize molecules and promote greater safety in various applications. Natural product chemistry is based on the science of degrading a molecule to known smaller molecules via known chemical processes and conforming the assigned structure through chemical synthesis from tiny, well-known molecules using well-established synthetic chemistry techniques.

Huge numbers of bioactive natural compounds with varied chemical structures have been found during the last century, and some have been investigated as therapeutic drugs to treat various disorders. Modern technology has aided in the development of natural product-based medicinal medicines. The synthesis of natural products not only allows for the confirmation of their molecular structures, but also provides the possibility for structural change to rationally optimize drug-likeness criteria and evaluate the bioactivity of analogues, exceptionally when inorganic and organic chemistry do not synthetize natural-like compounds. There is the hypothesis, that seaweed targeted compound can be extracted from natural raw source with control. During the extraction, isolation and/or purification, this compound can chemically modify to be safe or giving new features, like methylcellulose (normally used in the sports drinks and eyedrops), from bacteria, microalgae, and plants, where modification processes involve functional group modification of the natural compound [95,96]. The most common example are the opioid and semi-opioid anesthetics. On the other hand, the use of modified compounds in pharmaceuticals is heavily reliant on their purity, degree of polymerization, molecular conformation, and homogeneity, all of which affect the final products’ performance in terms of solubility, particle size, viscosity, rheological characteristics, and so on.

Therefore, multi-, and transdisciplinary research initiatives including scientists from several disciplines and with overlapping and complementary knowledge are the most effective method to achieve innovative applications and products. The contemporary collaboration of chemists, biologists, and doctors during the drug discovery and development process is an excellent illustration of such teamwork. Their collaborative and integrated efforts should result in enhanced drug discovery and development practices that are more productive, less expensive, and have a higher chance of success in the clinic for drug candidates [97].

## 7. From Compound Targeting into Approval to Be Industrial Exploited Drug

Over the last 40 years, the complexity of drug development has expanded dramatically, necessitating a preclinical phase of drug research, an investigational new drug (IND) application, and extensive clinical testing before receiving Medicament/Drug Agency marketing clearance. In general, biologics license applications (BLAs) and new drug applications (NDAs) undergo extensive review processes prior to approval. Following approval, drug performance is resubmitted to regulatory agencies for post-marketing investigation. The goal is to promptly, after a thorough medical examination, deliver patients safer and more effective therapies [98].

**Figure 5:**
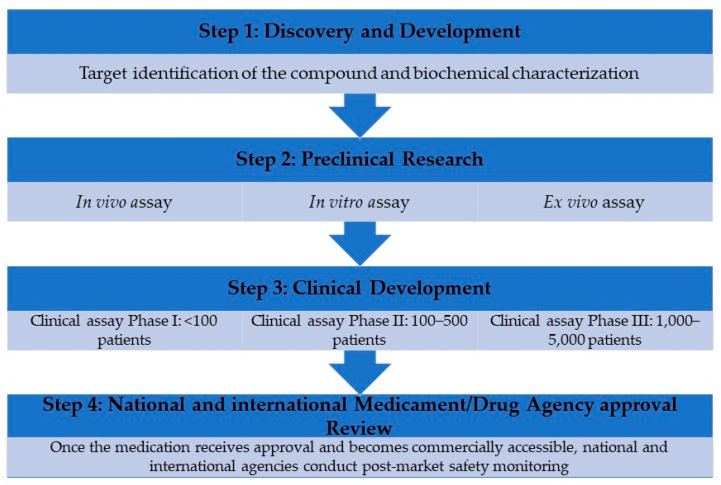
The stages of drug development.

The U. S. Food and Drug Administration (FDA) and European Medicines Agency (EMA) drug development process consists of five important components, each having several phases and stages. The stages of drug development are as follows in Figure 5.

### 7.1. Step 1: Discovery and Development

Drug discovery research is the process by which novel drugs are found. Historically, drug research, design, and development began with the identification of active components in traditional medicines or by coincidence. Afterwards, conventional pharmacology was used to examine databases of chemicals for natural compounds, tiny molecules, or extracts from plants and seaweed that have therapeutic qualities.

Disease processes, molecular compound studies, current medicines with unexpected side effects, and new technology all contribute to the drug discovery timeline shown below. Today’s drug discovery and development procedures include screening hits (where most of the seaweed compounds with positive hits are discovered), iterative medicinal chemistry, and hit optimization to decrease possible medication side effects (increase affinity and selectivity). Moreover, these steps in the drug development process boost the drug’s oral bioavailability, metabolic stability (half-life), and efficacy or potency.

Target identification is the discovery of a gene or protein (therapeutic agent) that plays an important role in illness. Drug targets must be effective, safe, and useful to fulfil clinical and commercial objectives.

Assay development is a critical component of the drug discovery pipeline. Assays are testing methods used to assess the effects of a novel drug candidate at the cellular, molecular, and biochemical levels.

Robots, data processing/control software, liquid handling equipment, and sensitive detectors are now used in High Throughput Screening (HTS) to run millions of pharmacological, chemical, and genetic tests in record speed, saving scientists hours of labor. HTS searches for active compounds, genes, or antibodies that bind to human molecules.

Pharmaceutical compounds with physiological activity that contribute to a drug candidate’s intended effects are known as active pharmaceutical ingredients, or APIs. All medications contain both the excipients and the API or APIs. Excipients are inert materials that facilitate the body’s absorption of medications. Compared to regular APIs, high potency APIs can be used at significantly lower dosage levels with comparable effectiveness. They are employed in intricate drug development processes including over 10 steps, and they are categorized according to their toxicity, pharmacological potency, and constraints on occupational exposure [98,99].

Once a primary chemical lead for a potential drug is identified, the focus of the drug discovery process goes further, signaling the initiation of the drug development stage.

### 7.2. Step 2: Preclinical Research

Once a lead molecule is identified, the preclinical phase of drug development begins with in vivo testing to establish the medicine’s effectiveness and safety.

Preclinical studies look at the new drug’s efficacy, toxicity, and pharmacokinetic (PK) data in nonhuman subjects. These investigations are carried out in vitro and in vivo with unrestricted dosages. Absorption, Distribution, Disposition, Metabolism, and Excretion (ADME) is a Pharmacokinetic (PK) procedure used to assess how a novel medication affects the body. Each impact is mathematically described in ADME. Proof of Principle (PoP) investigations are preclinical trials and early safety testing that are effective. In drug research and development programs, the terms proof of concept (PoC) and proof of principle (PoP) are practically interchangeable. Successful PoP/PoC investigations take the program to Phase II dosage studies.

In vivo, in vitro, and ex vivo tests are performed on entire, living creatures or cells, including animals, or on non-living organisms or tissue extracts. Examples of in vivo pre-clinical research include the discovery of novel medications utilizing mouse, rat, pig, and dog models. In vitro research is done in a laboratory. Using in vitro test systems alone cannot serve as the exclusive basis for screening chemicals, as there are significant differences in metabolism, bioavailability, and toxicokinetic between in vitro and in vivo test systems. Moreover, the absence of intercellular interaction and mechanisms related to endocrine homeostasis in in vitro systems poses a major limitation. Nonetheless, in vitro test systems can provide predictions regarding the potential of compounds to exhibit endocrine-disruptive effects. This implies that if substances do not show positive results in any of the in vitro tests, it is unlikely that they will function as endocrine disruptors in vivo. In vitro tests are thus employed in the ‘high-throughput screening’ of many chemicals [100].

Ex vivo research uses tissues or animal cells from a non-living animal. Ex vivo research investigations include, but are not limited to, the development of effective cancer therapy medications, the measurement of tissue properties (physical, thermal, electrical, and visual), and realistic modeling for novel surgical techniques. In an ex vivo test, a cell is always used as the basis for small explant cultures to provide a dynamic, controlled, and sterile environment.

Computer-based or computer-simulated test systems or biological investigations are known as in silico assays. As computational power and behavioral understanding of molecular dynamics and cell biology increase, these are probably going to become more widespread.

Oral, topical, membrane, intravenous, and inhalation are some of the new medication delivery techniques. Drug delivery systems are used to distribute new medications in a targeted or regulated manner. Physiological barriers in animals (or humans, in step 3) systems may hinder medications from reaching their intended target or from releasing at the appropriate time. The objective is to keep the medicine from interfering with healthy tissues while remaining effective.

Oral medicine administration is dependable, economical, and practical for patients. Although oral medication administration might not allow for precise quantities to be delivered to the appropriate location, it is suitable for preventive vaccines and dietary regimes. Delayed action, stomach enzyme breakdown, irregularities in absorption, or individuals with gastrointestinal difficulties or distress are all possibilities, and patients must be awake during treatment.

Ointments, creams, lotions, and transdermal patches are examples of topical drug delivery. Individuals with skin or muscular diseases prefer topical administration since it is non-invasive and allows them to self-administer the medication. Parenteral medication administration makes use of body membranes, such as intramuscular (IM), intraperitoneal (IP), or subcutaneous (SC). Usually, epithelial barriers are challenging for drugs to penetrate, and this method is commonly utilized in unconscious patients. One of the fastest ways to administer and absorb drugs is through intravenous injection. IV injection is more efficient than IM, SC, or LP membrane techniques because it ensures that all medicine dosages reach the circulation. By inhaling the medication, it is quickly absorbed into the mucosal lungs, nasal passages, throat, or mouth. Problems with inhalation delivery include patient discomfort and difficulties administering the right amount because of the small surface areas of the mucosa. Fine medication powders or mace are used in pulmonary inhalation drug administration macromolecular pharmaceutical solutions [98,99].

Throughout the pre-clinical and clinical stages, formulation optimization is continuous. It ensures that medications are administered to the correct location at the correct timing and concentration [101]. Optimization may involve addressing solubility issues.

### 7.3. Step 3: Clinical Development

Preclinical research is followed by clinical drug development, which entails volunteer studies and clinical trials to modify the medication for human consumption. Research done during this phase may be impacted by the complexity of Clinical Trial design, the associated costs, and the implementation issues. Trials must be conducted in a way that guarantees the medication performs as best it can for its intended purpose, be safe and effective, and stay within the drug development budget. This rigorous process needs to be set up correctly and involve a lot of volunteers to be successful. Appropriate dosage determines the effectiveness of medication; clinical trials assess dose escalation, single ascending, and multiple dose studies to find the appropriate patient dosage. This is the first time the medication (Clinical assay Phase I) has been tested on humans. Less than 100 volunteers will help researchers evaluate the drug’s safety and pharmacokinetics, as well as its effects on the body’s absorption, metabolism, and excretion, as well as any negative effects for safe dose ranges.

Assay intended for clinical application Phase II assesses the safety and effectiveness of the treatment in an additional 100–500 patients, who may receive a placebo or a conventional pharmaceutical that has been used as therapy in the past. Scheduling is aided by the study of the proper dose strength, and side effects and risks are documented. Phase III allows for prescription labeling and appropriate drug usage instructions, with enrollment ranging from 1000 to 5000 patients. In preparation for full-scale manufacturing after medication approval, phase III studies require extensive planning, coordination, and oversight by the Institutional Review Board or Independent Ethics Committee. When conducting clinical trials, testing facilities collect, store, and distribute biological samples in compliance with international standards and regulations. Different biological samples fall under different categories. Molecular markers of a drug’s action on the intended human region, known as pharmacodynamic (PD) biomarkers, link the treatment plan to physiological responses. This information may be used to pick sensible combinations of targeted agents as well as optimize treatment regimens and timetables. The inclusion of PD endpoints in human studies improves rationality and hypothesis-testing power. Pharmacokinetic (PK) Analysis is an experimental study that evaluates how a new medicine performs in the human body. Compartmental modelling is used to determine the volume of distribution, clearance, and terminal half-life.

Bioanalytical procedures identify analytes and metabolites in biological or human samples, such as drug or biomarkers, to estimate drug efficacy and safety. The entire bioanalytical test includes sample collection, cleaning, analysis, and detection. Stability is critical in assessing the efficacy of human drugs, and biological samples are necessary. Because drugs and their metabolites break down easily, drug concentrations may drop during the duration of a drug’s life course [98,99].

In 2008, a clinical study was authorized to assess the effectiveness of carrageenan-based gel as a preventive measure against sexual HIV infection in women [102]. 6202 HIV-negative, sexually active women 16 years of age and older participated in this experiment and were followed up with for up to two years. Random assignments were made to give participants a placebo or a carrageenan gel therapy (n = 3103). Although no safety problems were mentioned, the results did not support the efficacy of carrageenan-based gel in preventing HIV transmission from male to female. The results could have been impacted by the gel’s less-than-ideal adherence, as it was rarely used during sex. Despite this negative result, research into female-controlled HIV prevention strategies needs to continue to understand the barriers that prevented carrageenan from being a more effective anti-infection agent. 400 clinically healthy professionals, including doctors, nurses, kinesiologists, and other medical staff, who had direct patient interaction with COVID-19 hospitalized patients participated in a clinical investigation [103]. For 21 days, either ɩ-carrageenan nasal spray or a placebo was administered to these subjects four times a day. The principal outcome evaluated was a clinical illness verified by reverse transcriptase–polymerase chain reaction (PCR) identification of SARS-CoV-2. Through a Google Form survey, patients used a standardized questionnaire to track their symptoms daily. The center’s investigator determined if there was any other possible explanation for the symptoms except COVID-19. Individuals exhibiting questionable symptoms were isolated prophylactically until the PCR result was obtained after undergoing a nasopharyngeal swab with a PCR test for SARS-CoV-2. 48 h after symptoms subsided, those who tested negative for the virus returned to their place of employment, while those who tested positive for the virus remained in isolation to manage the illness. Participants with a negative PCR but persistent symptoms after 48 h underwent a new PCR. Results revealed that six participants were excluded from the final analysis due to suggestive COVID-19 symptoms at randomization. Among the remaining 394 participants (196 in the iota-carrageenan group and 198 in the placebo group), thirteen individuals from both groups withdrew consent before the specified day. Forty-three participants underwent a PCR test due to COVID-19 suggestive symptoms, with 31 testing negatives (7.6% in the iota-carrageenan group and 8.6% in the placebo group). During the 21-day follow-up, 12 out of 394 individuals (3.04%) developed new COVID-19 cases (symptomatic with confirmed PCR). The incidence of COVID-19 changed considerably between those who received the -carrageenan nasal spray (2 of 196, 1.0%) and those who received the placebo (10 of 198, 5.0%), indicating that carrageenans might be an effective preventive medication for avoiding SARS-CoV-2 infection.

Furthermore, clinical studies demonstrated the antidiabetic properties of carrageenan. In a study with participants, those who ate porridge with λ-carrageenan showed a significant reduction in post-prandial glycemic reactions as compared to the control group. This suggests that carrageenan may help manage metabolic diseases like diabetes when paired with dietary fiber [104].

During a clinical investigation, Power Fucoidan Cream^TM^ (4% fucoidan cream) using fucoidans extract from *Nemacystus decipiens* (Phaeophyceae) was used to treat two patients with recurrent oral herpes labialis (ROHL), which is frequently brought on by herpes simplex virus type-1 (HSV-1). After one week of topical treatment, the infection significantly improved in terms of healing and pain reduction [105]. Commonly, antiviral drugs such as oral acyclovir (Zovirax^TM^ 400 mg tid × 7 days), oral valacyclovir (Valtredx^TM^ 500 mg bid × 5 days), topical acyclovir (5% Zovirax Cream^TM^), and topical vidarabine (3% Arasena-A Cream^TM^) have been used for ROHL. Although Power Fucoidan Cream^TM^ [106] has been investigated from the perspectives of cosmetics and skin-care agents, few studies have focused on oral disease. The study originally looked at the use of PFC for the treatment of symptomatic ROHL in two typical clinical instances involving discomfort when eating and speaking that had proven resistant to numerous treatments. The results of topical PFC application were astounding. The outcomes of PFC treatment in just two individuals are difficult to interpret since the reported responses might simply represent the normal course of the disease rather than the effects of medicine. Although the processes are not fully understood, it is quite likely that fucoidan cream had actual therapeutic effects because previously persistent lesions did not return. As a result, research trials are needed to determine the clinical efficacy, usefulness, and safety of topical fucoidan cream as a therapy for ROH.

### 7.4. Step 4: National and International Medicament/Drug Agency Approval Review

Numerous factors, such as toxicity, effectiveness, PH characteristics, bioavailability, and subpar drug performance, might cause new drug applications to fail. A novel treatment may be rejected because of safety concerns about its use after production if its toxicity in humans or animals is too high. The medicine/drug authorities have the right to reject a novel medication if the evidence is unclear or the medicine’s efficacy is not strong enough. An inability of a medication to pass the evaluation of medication/drug authorities may also be caused by poor bioavailability resulting from low water solubility or extensive first-pass metabolism. Unexpected human medication interactions and inadequate action duration are two PK reasons for pharmaceutical failure. The medication/drug authorities may reject the application if the new medication only partially fulfills the necessary purpose, in favor of a better formulation [98,99].

Thus, it is still a long road from isolating a seaweed compound until the further commercial application.

If the medicament is approved and commercially available, there is a post-market safety monitoring by the national and international agencies.

### 7.5. Regulatory Considerations

Drug development entails producing ever bigger quantities of medicine, and modifications in methods for different-sized batches may result in unforeseen complications. The usage of the proper pharmaceutical equipment, as well as the identification of factors that impact key process parameters, can be beneficial [98,99].

The process of developing new drugs is highly regulated and complex, requiring the knowledge and advanced research and development skills of the medical research community. Clinical trial participants, whether human and animal, must be handled with the utmost professionalism and care, and all laws and safety precautions must be followed to the letter. The goal of drug development is to prevent suffering in humans and animals whenever possible. It also aims to find and provide novel treatments that can be used to enhance human health and well-being.

Seaweed extracts may be obtained and used in a variety of secondary products, including antibacterial, antifungal, antiviral, and anti-inflammatory medicines. By doing so, the health of people and animals may be improved by reducing reliance on chemical products and related resistance problems, such as antibiotic resistance. Additionally, seaweed extracts exhibit antiviral action against a variety of viruses, including coronavirus, either indirectly by boosting the host’s immune system or directly by having a virucidal effect and blocking virus attachment receptors [39].

Reactive oxygen species are produced when human skin is exposed to environmental stressors such as sun radiation, pollutants, and chemical ingredients found in cosmeceuticals. These factors can lead to a range of skin-damaging issues, including wrinkles, dark circles, dullness, and age spots, as well as carcinogenesis. Seaweed-based cosmetic products can act as natural alternatives to synthetic chemicals, and bio-purified seaweed components have proven to be particularly successful in these formulations. As bioactive ingredients for antiaging, anti-acne, deodorizing, antimicrobials, antioxidant, moisturizing, whitening agent, anti-wrinkle, anti-inflammatory, sensory enhancer, UV protection, anti-allergic, stabilizer, viscosifier, and thickeners, primary and secondary seaweed metabolites can be found in cosmetic products.

However, monitoring the biochemical properties of seaweed-based extracts remains an unresolved challenge. Product safety and customer protection must be prioritized in each new product. Seaweed-based products can be used to make traditional herbal treatments (traditional medicine) [39].

Pharmaceutical drugs containing seaweed components must conform with EU medicine law in the EU. Directive 2001/83/EC establishes the EU rules for human medicines. The following is the definition of a medical product:

“(a) Any substance or combination of substances presented as having properties for treating or preventing disease in human beings; or

(b) Any substance or combination of substances which may be used in or administered to human beings either with a view to restoring, correcting, or modifying physiological functions by exerting a pharmacological, immunological, or metabolic action, or to making a medical diagnosis” (Directive 2001/83/EC, Title I, Article 1).

The European Medicines Agency evaluates the evidence, and the EU Commission grants the license in the centralized procedure. The national procedure, the mutual recognition procedure, the de-centralized procedure, and the centralized procedure are the other four methods used to authorize pharmaceutical drugs. For further details, visit the European Medicines Agency’s webpage on these procedures. In addition to the “normal” regulatory procedure for synthetic medicines, the Directive 2011/83/EC specifies specific processes for (a) herbal medicinal products and (b) traditional herbal medicinal products. Algae are components of herbal compounds, and algal preparations and substances are herbal medicines.

The end products of procedures like extraction, distillation, or fermentation are herbal preparations. The approval of herbal medicinal products requires scientific proof of established therapeutic usage, acknowledged effectiveness, and a manageable level of safety. The vendor must only show traditional use for the product to be recognized as a traditional herbal medicine; no therapeutic claims may be made. Conventional use is defined as 30 years or more of continuous use, of which at least 15 years are spent inside the EU [107].

A product that is offered as a dietary supplement yet contains a health-improving ingredient derived from seaweed is allowed to be sold. While medications treat or prevent illness, foods may claim to promote health or reduce the risk of disease. If a product contains a specific quantity of a medicinal plant for it to serve a therapeutic purpose, it is classified as a medication. The classification is determined by the dose. Products that are not clearly labeled as food or medications need to be handled as such. Marketers should consult the authorities for a classification decision if they are unclear whether a product qualifies as a pharmaceutical based on its intended use. Although national medical authorities classify products on their markets (food vs. medicine), there is a significant disparity between them. Based on the two limbs of the EU legal definition of medicine, a product is automatically qualified as a medication if it has a pharmacological influence and presents therapeutic claims. This suggests that a product cannot be marketed as food if it claims to have medicinal qualities. Foods cannot claim therapeutic benefits. Regulation EC/1924/2006 governs nutrition and health claims for foods (including food supplements) at the EU level. EFSA assesses the scientific data used to support these claims. Nutritional claims are permitted provided they are included in the Regulation’s Annex. The guidelines for food supplements are the same as they are for regular foods.

Macroalgae have numerous features that make them appealing for the development of novel products. Human health and consumer rights should be safeguarded by substantive and procedural rules pertaining to products, without placing unnecessary restrictions on businesses in Europe or other countries. EU directives set common objectives for all member states, but each country is free to decide how to get there. Inconsistencies may arise from directives. EU regulations, on the other hand, take effect instantly in member states and do not need to be implemented at the national level. All parties can see a clearer working environment thanks to harmonized EU regulations. The establishment of centralized processes for novel foods, food additives, medications, cosmetics, and fertilizers/bio-stimulants, along with the creation of an EU-wide methodology for evaluating product safety, contribute to the realization of the product single market [107].

### 7.6. Seaweed Pharmacodynamics and Pharmacokinetics: A Step into Clinical Assays

Pharmacodynamics and pharmacokinetics are determined by the bioavailability of the seaweed element and how it is absorbed, passed through the colon, and into the circulatory system after a meal. Therefore, a variety of mechanisms, including as drug matrix liberation, absorption, distribution, metabolism, and elimination, are involved in bioavailability. Seaweed constituents can be consumed in complicated combinations or as single, pure substances. Gastric acid from the stomach may cause early alterations to the therapeutic drugs during the absorption process. Additionally, medications may cleave in the small intestine after ingestion, releasing the active ingredient radical. Additional seaweed compound structures may also engage in conjugation processes leading to the formation of sulphate, glucuronide, or methyl groups [77]. When seaweed compounds are absorbed, they usually go through one of four routes: (1) excretion in the feces; (2) absorption by the colon or intestinal mucosa, which is followed by entry into the portal vein for liver delivery; (3) further conjugation in the liver, which may lead to the addition of methyl, glucuronide, or sulphate groups, and then release into the bloodstream for tissue absorption; (4) excretion in the urine. Though the physical and chemical characteristics of the bioactive compounds play a major role in determining the absorption kinetics, the subject’s physiology (including age, gender, genetic pro-file, and lifestyle) can also have an impact, producing a unique bioavailability profile. Chemical half-lives can therefore vary from minutes to hours [62,63,77].

As a result of these activities, there is growing attraction in use of seaweed compounds as treatment for a variety of diseases (mostly, phenolic compounds and sulphated polysaccharides). However, there are several challenges to using marine polyphenols as a therapy. Identifying and isolating specific seaweed compounds with therapeutic potential is one of the challenges [57,58]. As seaweeds contain a wide variety of chemicals, it might be challenging to identify certain molecules with potential for medicinal use. Additionally, the extraction and purification of seaweed components generally lack standardized procedures, which may have an impact on the finished product uniformity and quality [108]. Another barrier is a lack of understanding of the pharmacokinetics and pharmacodynamics of seaweed compounds. Unlike manufactured pharmaceuticals, seaweed chemicals have intricate structures that might impact their distribution, metabolism, excretion, absorption, and bioavailability. It might be challenging to determine the ideal dosage and frequency of administration of seaweed compounds due to their complexity [109]. Furthermore, the isolated biomolecule’s appropriateness for industrial and medicinal uses should be validated by a suitable clinical evaluation with a safety report included. Recent breakthroughs in metagenomics, genomics, proteomics, molecular biology, and bioinformatics assays may help much in the discovery of novel medications from seaweeds [39].

Pozharitskaya et al. [110] explored the pharmacokinetics following the topical administration of fucoidans derived from *Fucus vesiculosus* (Phaeophyceae) in skin rat. The findings indicate that following topical therapy, a significant portion of the fucoidan-based drug was trapped in the epidermis and gathered in the striated muscle. Topical administration of 50–150 mg/kg single doses and 100 mg/kg repeated doses over five days did not result in rat skin erosion/excoriation, erythema, hemorrhage, or edema. Obluchinskaya et al. [111] also delved into the kinetics of topical fucoidan application to evaluate the anti-inflammatory effect of fucoidan isolated from *Fucus vesiculosus* in rat paw edema produced by carrageenan. After topical application, the fucoidan-based cream prevented paw edema and showed efficacy at levels comparable to those of diclofenac gel, a synthetic anti-inflammatory drug used to relieve pain and inflammation. The results suggest that topical use of a fucoidan-based cream could be a useful treatment for skin problems and inflammation, as fucoidan release was found to be controlled by drug diffusion into the skin. Consequently, a comprehensive understanding of pharmacokinetics provides valuable insights for elucidating the molecular basis of pharmacological activity, adjusting doses and treatments, and achieving more targeted drug applications.

Despite these challenges, there are lots of chances to use seaweed compounds as medicine. The creation of novel treatments for illnesses for which there are now few available options is one of the opportunities. Another chance is to create new goods for the food and cosmetics sectors [77].

### 7.7. Formulation Strategies and Delivery System

The initial stages of exploring seaweed polysaccharides for drug delivery applications are underway [112]. Seaweed is a frequent source of polysaccharides such as alginate, carrageenan, fucoidan, ulvan, and laminarin. These natural polymers may be converted to nanoparticles (NPs) via a variety of processes such as ionic gelation, emulsion, and polyelectrolyte complexing. Ionic gelation and polyelectrolyte complexing involve the addition of cationic molecules to anionic polymers, resulting in NPs with desirable form, size, and charge properties. Seaweed polysaccharide-based NPs have been prepared using various techniques, and their use as carriers for delivering therapeutic molecules such as proteins, peptides, anti-cancer medicines, and antibiotics has been investigated. Seaweed polysaccharide-based NPs have a desirable particle size, good drug encapsulation, and long-term drug release, as well as high biocompatibility. This highlights their considerable potential for safe and efficient medication delivery.

Derived from seaweeds, hydrocolloids find broad applications across various industries, serving as gelling agents, coatings, stabilizers, and ingredients in cosmetics. Investigating the advantageous properties of these seaweed-derived compounds presents possibilities for crafting targeted functional foods and medical products to meet diverse requirements. Given their demonstrated therapeutic activities, it is of utmost importance to devise innovative systems that incorporate phycocolloids in the creation of new medical products. Literature consistently highlights the utility of carrageenan, alginate, and agar for pharmaceutical aims, emphasizing the ongoing exploration of novel drug delivery systems [1,2,3,21,55,64,76,80].

Furthermore, the combination of carrageenan and alginate with other natural polymers (per example, chitosan and cellulose) shows potential for producing biodegradable materials suitable for secure biomedical uses. Alginates, being non-toxic hydrogels with remarkable biocompatibility, are well-suited for various applications in the pharmaceutical industry. They serve as auxiliaries in drug delivery, tissue healing, and alleviating symptoms associated with esophagitis and heartburn [113,114,115].

The investigation carried out by Alves et al. [116] illustrated the utilization of ulvan hydrogel for drug delivery. The crosslinking of ulvan with 1,4-butanediol diglycidyl ether, along with the inclusion of the drug molecule dexamethasone, resulted in a gradual and sustained release over a 14-day period.

The algae business is actively seeking natural sources of useful and rich components generated from sustainable and cost-effective raw materials, such as phlorotannin, fucoidans, laminarin, fucoxanthin, carrageenan, alginate, and others. These ingredients have the potential to drive innovation in the food and cosmetic sectors. Despite the fact that algae are a readily available renewable resource, many brown seaweeds are underutilized, mostly being turned into fertilizers and animal feeds. More research is needed to better understand the effects of seaweed production and exploitation on safety, toxicity, and environmental problems. The development and marketing of phlorotannin-containing products is hampered by difficulties in generating them on an industrial scale and at reasonable costs, as well as a lack of thorough understanding about their entire chemical characterization. This understanding is critical for substantiating the probable biological activity revealed in recorded in vitro experiments. Pre-processing activities, extraction techniques, long-term storage, food manufacture, and digesting conditions all have important roles in either increasing or decreasing phlorotannin concentration, and therefore determining health benefits [117].

Furthermore, phlorotannins go through several metabolic processes. These metabolic pathways, like terrestrial polyphenols, may be divided into three stages. Intramolecular changes such as oxidation, reduction, and hydrolysis dominate Phase I processes. Phase II processes, on the other hand, include the conjugation of endogenous molecules with ingested chemicals, transforming them into water-soluble substances readily eliminated from the body—a detoxification process accomplished by acetylation, methylation, glucuronidation, and sulfation. Notably, in the context of drug or food metabolism, the phrase “Phase III reaction” has received considerable attention since it is responsible for the ultimate clearance of poisons and metabolic products from cells. These processes significantly affect the phlorotannins’ stability during transit [118].

However, ensuring the stability of phlorotannins poses a critical challenge, as factors related to storage can have adverse effects on these delicate molecules. Consequently, preservation strategies must be carefully chosen to guarantee the stability of phlorotannins and their associated health benefits. Research has outlined the most recent applied approaches to mitigate the impact of technological processes—ranging from preprocessing to passage through the gastrointestinal tract on phlorotannin stability [117]. Noteworthy findings regarding the stability of phlorotannins during pre-processing, extraction, and storage include the following: immediate freeze-drying of algae material (devoid of impurities) after harvesting, followed by storage in vacuum-packed packages at freezing temperatures, can extend the algae’s shelf life by reducing moisture content and minimizing spoilage before extraction. It is observed that minimizing pre-treatments leads to better maintenance of phlorotannin content, considering various parameters that can affect extraction efficiency, such as solvent type, extraction time, temperature/pressure, solvent-to-solid ratio, and the particle size of dry seaweed. Optimization techniques that assess the impact of these influencing elements and their interplay on extraction yield are desperately needed. Encapsulation appears to be a potential technique for generating stable goods, since it provides protective mechanisms to keep phlorotannins in their active molecule state throughout storage till consumption. More study is needed to develop sustainable seaweed processing methods that provide cleaner exploitation, simpler industrial scale-up, increased resource efficiency, and the generation of co-products rather than waste, in accordance with Green Chemistry principles.

To conclude, the medicinal implications of hydrocolloids and other derivatives from seaweeds requires robust research support. Ongoing research and development in this area could lead to major advancements in a range of biomedical applications.

### 7.8. Seaweed Approved Drugs

A depsipeptide called Kahalalide F, derived from the green seaweed *Bryopsis* sp. (Chlorophyta), has been trademarked is being investigated in cutting-edge clinical trials for the treatment of liver cancer and for usage in lung cancer in humans [2,28,119]. In contrast to traditional therapy, patient response was satisfactory without producing acute reactions to treatment [2,28,119].

In clinical stage research with human patients, an old chemical (Algasol T331) based on brown seaweed polymer was used in cancer treatment. This substance was found to be effective in cancer patients’ post-surgery and post-radiotherapy treatment. The primary benefits of compounds were that they shielded the body and sped up its recovery from more potent and damaging cancer treatments, namely in terms of reducing asthenia and restoring hematic function [120].

Recently, China’s authorities had authorized a medication to treat Alzheimer’s disease, the new therapy with the possibility to cure the cognitive ailment in 17 years. According to a statement from China’s medication safety regulator, Oligomannate, a seaweed-based treatment, can be used to treat mild to moderate Alzheimer’s disease [121].

In cardiovascular disease, the aim of seaweed phlorotannin supplements are to prevent arteriosclerosis and increase protective high-density lipoprotein cholesterol (HDL-C). Phlorotannin-containing products include HealSea^TM^ (manufactured by Diana Naturals in Rennes, France), IdAlg^TM^ (produced by Bio Serae in Bram, France), and Seanol^TM^ (produced by LiveChem in Jeju-do, South Korea and distributed by Simple Health in Maitland, FL, USA). InSea2^TM^ (Rimouski, QC, Canada), a commercial mixture of phlorotannins from *Ascophyllum nodosum* and *Fucus vesiculosus* (Phaeophyceae), supports a 90% reduction in postprandial blood glucose levels [16]. However, they are considered nutraceutical solution and not medicament or official medical therapeutics.

Determining bioactive substances with anti-cancer properties or the capacity to be employed in innovative treatments is a long way from the lab to real-world utilization. Mostly, due to seaweed ability to change almost all the compounds, which alters all the stability to exploit them commercially. In other hand, there is new innovations which can be a key into the greatest potential of the seaweed in the medicines.

## 8. Future Perspectives and Challenges

To evaluate the potential of seaweed as a novel source of medicines, with the goal of developing innovative medications with natural origin ingredients and reducing collateral effects caused by synthetic compounds. Less than half of the world’s population today considers access to essential medicines a luxury because of the high cost of pharmaceuticals and the low purchasing power of people in various continents, such as Asia and Africa [3,16].

Traditional medicine is this community’s first line of defense when it comes to treating health issues because it suggests using natural ingredients like plants, herbs, and seaweeds to treat illnesses and difficulties. Due to this, traditional medicine and modern treatment can coexist, as is the case in developed countries like Australia, Canada, and France. However, traditional medicine does not adequately characterize the biochemical properties of seaweed extracts, which might have unfavorable effects like the recurrence of allergies or the assimilation of toxic compounds that can damage our bodies. Several studies on seaweed compounds and extracts have been completed; however, further research must be conducted to go deeper and identify unique molecules to incorporate in various biotechnological uses and applications, thereby openly and incidentally enhancing human welfare [3,16].

Seaweeds may be used in a variety of diverse applications due to their unique qualities. For example, bioactive substances that are isolated from seaweed can be used to make products that are antimicrobial, antioxidant, anticancer, antifungal, antiviral, anti-inflammatory, and antioxidant. By lowering reliance on chemical products and associated resistance problems, like antibiotic resistance, the use of seaweed in medicinal products may improve the health of people and animals. Reactive oxygen species are formed more frequently when human skin is overexposed to UV radiation, pollution, and chemicals used in cosmeceuticals. This can lead to a variety of skin issues. Seaweed-derived purified metabolites have the potential to be utilized in synthetic cosmetics and medications as natural anti-aging, anti-acne, deodorizing, moisturizing, whitening, anti-wrinkle, anti-inflammatory, sensory enhancer, UV protection, anti-allergic, stabilizer, viscosifying, and thickening agents [39].

Seaweed growers need the cooperation of policymakers to manage market demand and get beyond obstacles including financial, policy, and planning constraints. Among the industrial barriers are a lack of knowledge about the use of seaweed in a range of domains and the absence of a thorough national and international seaweed strategy. Seaweeds need to be used worldwide, and greater knowledge from Asian nations that are leaders in seaweed production and cultivation needs to be acquired. Seaweed productivity can be quickly determined by applying seaweed for climate change mitigation. To boost the production of seaweed and the value of seaweed products, future research must identify seaweeds as bioactive chemical components and alternative diets. While looking for affordable ways to solubilize important biomolecules and use bioactive compounds as natural contributions in a variety of fields, researchers must also highlight the limitations of bioactive component extractions. Examine how seaweed habitats sequester carbon and how much carbon is released overall during the seaweed production process. A carbon-sequestration-based assessment of the seaweed life cycle, from cultivation to consumption, is also necessary [39].

As a result, multi- and transdisciplinary research projects involving scientists from several disciplines with overlapping and complementary expertise are the most effective technique for developing breakthrough applications and products. The current collaboration of chemists, biologists, and clinicians during the drug discovery and development process is a good example of such collaboration. Their integrated and coordinated efforts should result in improved drug discovery and development practices that are more productive, less expensive, and have a greater likelihood of success in the clinic for drug candidates [97].

## 9. Conclusions

The current study explains the whole process that leads to a seaweed extract being recognized as a medicinal agent and becoming commercially accessible on the market. Companies interested in investing in the seaweed economy must embrace biology-based management. This necessitates the use of ecologically friendly techniques, such as collecting and processing seaweeds in accordance with their life cycle and bioavailability. Moreover, it is imperative to equip seaweed growers with scientific advisors to identify biological traits of seaweed and to increase yields through training and equipment. Despite its multiple medicinal applications, seaweed supplementation and commercialization as food or food products, as well as pharmaceutical possibilities, have been unsuccessful. This review also demonstrates that there is road for the chemical medicine, biochemistry, and similar areas to study the seaweed compounds and furthermore, trying to convert natural molecules into stable and efficient compounds. This study highlights new trends and important knowledge that can be key to surpass the seaweed compounds finding into a drug applied to humans, where it is important to have a team with (bio)chemical, pharmaceutical/biomedical background associated with (marine) biology/biotechnology groups. As demonstrated above there is a long way until a seaweed compound can successfully be considered a commercial approved drug.

Only a few commercial seaweed products are now available; so, coordination between research and industry is crucial for their increased utility. As a result, employing seaweed chemicals as a therapeutic has both advantages and disadvantages. Despite these difficulties, research on seaweed compounds is intriguing due to their potential benefits in treating a variety of illnesses and creating new goods. To overcome the challenges and completely realize the medicinal potential of seaweed compounds, more research is required.

## Figures and Tables

**Table 1:** Methods and techniques to identify, quantify and isolate seaweed’s compounds.

Technique	Equipment	Target Compound	Reference
Chromatography	Liquid chromatography	Pigments, phenolic compounds, fatty acids, amino-acids	[49,50,51]
Spectrophotometry	Ultraviolet/visible spectrophotometer	Pigments, phenolic compounds, proteins	[52]
Spectroscopy	Fourier-transform infrared spectroscopy	Polysaccharides, pigments, phenolic compounds, microplastics	[53,54]

## Data Availability

Not applicable.
